# Clinical Presentations and Nosocomial Infections of Neurolisteriosis

**DOI:** 10.1155/cjid/5960643

**Published:** 2025-06-12

**Authors:** Javier Vicente Trejos Pino, Juan Carlos Rodriguez Delgado

**Affiliations:** ^1^Department of Infectious Diseases, Guayaquil National Police Teaching Hospital, Catholic University of Santiago de Guayaquil, (Universidad Católica de Santiago de Guayaquil), Guayaquil 090510, Ecuador; ^2^Radiological Studies and Diagnostic Imaging Center of Ecuador (CERID Ecuador), University of Guayaquil (Universidad de Guayaquil), Guayaquil 090510, Ecuador

**Keywords:** hospital-acquired, immunocompromised, listeria brain abscess, listeria meningoencephalitis, rhombencephalitis, RTE food

## Abstract

Neurolisteriosis is a listerial invasive disease, which is characterized by brain parenchymal and meningeal involvement, with a high fatality rate and frequent neurological sequelae. The main clinical presentations of neurolisteriosis are meningitis, meningoencephalitis, rhombencephalitis, and brain abscess. Neuroradiological imaging is useful to distinguish these clinical presentations. The diagnosis of neurolisteriosis may be confirmed by cerebrospinal fluid or blood cultures, but these tests may have different yields depending on the clinical presentation of neurolisteriosis. The elderly and immunocompromised patients are the most susceptible population to developing neurolisteriosis, and few cases occur in healthy young people. This disease is caused by *Listeria monocytogenes*, a foodborne pathogen with an intracellular life cycle, which can be found in processed foods, and it remains the third cause of bacterial meningitis in adults. Most cases of neurolisteriosis are community-acquired, but several hospital-acquired cases and outbreaks have been reported in the literature and linked to the consumption of food served to inpatients. Aminopenicillins are the antibiotics with the highest impact on the prognosis of neurolisteriosis, and alternative antimicrobial therapies must be considered in those cases where a first-choice antibiotic cannot be administered or with antibiotic treatment failure. In this article, the epidemiology, sources of infection, pathogenesis, and clinical aspects of neurolisteriosis are reviewed, highlighting the main clinical presentations of the disease. Relevant information regarding hospital-acquired neurolisteriosis is also included to provide a framework for discussing nosocomial cases definition.

## 1. Introduction

*Listeria monocytogenes* is a foodborne pathogen found in soil, water, and feces of animals and humans [1, 2]. This pathogen can cross the intestinal barrier, the blood–brain barrier (BBB), and the fetoplacental barrier, infecting organs such as the brain or uterus, thereby causing severe life-threatening infections, called listeriosis or invasive listeriosis [2–4]. The commonest syndromes of invasive listeriosis (meningitis, meningoencephalitis, and septicemia) mainly affect individuals with impaired cell-mediated immune response causing mortality rates ranging from 17% to more than 30% [5–8]. Invasive listeriosis rarely occurs in healthy adults; in these individuals, the infection can cause self-limited febrile gastroenteritis [1, 3, 6]. Neurolisteriosis is a central nervous system (CNS) invasive listeriosis that comprises four main clinical presentations, such as meningitis, meningoencephalitis, rhombencephalitis, and brain abscess; nonetheless, listerial meningoencephalitis is the predominant clinical presentation [1, 2, 5, 7, 8].

Foodborne acquisition of listeriosis among inpatients has been well documented through case series and outbreaks [2]. Given that most cases of neurolisteriosis are community-acquired, the diagnosis of hospital-acquired neurolisteriosis may be unsuspected. Furthermore, confirming the source of nosocomial infection could be challenging, as it requires complex logistics to search for the presence of *L. monocytogenes* in foods consumed by patients during their hospital stay. We present a review of the clinical presentations of neurolisteriosis in the nonpregnant adult population to distinguish their diagnostic and therapeutic approaches. Available information on nosocomial cases is also described, prompting clinicians to maintain suspicion of *L. monocytogenes* in patients with a history of immunocompromise who develop acute meningoencephalitis signs during a hospital stay, which also encourages promoting preventive measures for this pathogen in healthcare settings.

## 2. Epidemiology

*Streptococcus pneumoniae* and *Neisseria meningitidis* are the most common pathogens of community-acquired bacterial meningitis (C-ABM) in adults, causing 71% of all cases worldwide [9]. After the vaccine-related decline in *Haemophilus influenzae* type B meningitis, *L. monocytogenes* has become the third most frequent cause of C-ABM in adults, found in approximately 5% of cases [7–9]. Nevertheless, this low incidence of *L. monocytogenes* C-ABM contrasts its mortality rate, which ranges from 17% to more than 30%, even when appropriately treated [5–12]. A meta-analysis found a higher global case fatality ratio (CFR) for *L. monocytogenes* C-ABM (CFR 27%) than that reported for pneumococci (CFR 24%), *H. influenzae* (CFR 11%), and meningococci (CFR 9%), with a similar trend in the adult population [9]. However, most of the neurolisteriosis data reported in the literature come from statistical information from high-income countries. Therefore, the incidence and mortality of neurolisteriosis worldwide may be significantly underestimated due to missing data from roughly 48% of the global population, mainly in developing countries [9, 12]. For instance, in Ecuador, a low-income South American country, listeriosis is not a notifiable disease, and there is no National Surveillance Program for *L*. *monocytogenes* [13–15]. Furthermore, there is a lack of case-reported publications about neurolisteriosis in this country. Nevertheless, contamination sources of *L. monocytogenes* found in food products marketed in many provinces of this country have already been published [13–16].

Hospital-acquired listeriosis has been documented in case series and outbreaks reported in the last 40 years in persons who were, or had recently been, hospitalized for another reason and had consumed food in healthcare facilities [17–20]. Data are lacking on the global or regional epidemiology of healthcare-associated neurolisteriosis per se, but epidemiological information about nosocomial invasive listeriosis, including neurolisteriosis and listerial septicemia has been described. In this regard, two literature reviews reported a high proportion of nosocomial cases among all types of listerial disease [18, 20]. A worldwide review found that 16% of 369 adults with listeriosis were hospital-acquired, and most had a CNS infection [18]. A larger review of invasive listeriosis (meningitis and bacteremia) that included 13 case series from the United States, European countries, Australia, and China found that roughly 25% of 840 cases reported were hospital-acquired [20]. In the same review, the cases and outbreaks reported with cerebrospinal fluid (CSF) culture positive for *L. monocytogenes* (confirmed neurolisteriosis) were from high-income countries, such as the United States of America, China, Germany, and Norway [20]. Thus, as with community-acquired cases, there is a lack of available data concerning nosocomial cases from developing countries as well.

## 3. Source of Infection

This foodborne pathogen is frequently isolated from several food products and food-processing environments owing to its resistance to environmental stress, ability to multiply at low temperatures, and capacity to form biofilms on the surfaces of different sites in food-processing facilities [2, 3, 10, 21]. These food products known as ready-to-eat (RTE) foods are common vehicles involved in listeriosis cases and outbreaks [3, 10, 21]. Since this bacterium is inactivated by heat treatments, RTE foods at risk of *L. monocytogenes* contamination are usually raw foods that are subjected to minimal further processing, such as soft cheeses, sausages, RTE meat products, raw meat (especially turkey and chicken), pâtés, any meat conserved after having been heated, seafood (particularly salmon and mussels), sandwiches, vegetables, fruit juice, fruit salad, and raw milk or products made from this ingredient [6, 10, 13–16, 21, 22]. Nevertheless, it has been described that healthy people in a high-income country consume between 10^6^ and 10^9^ colony-forming units (CFU) of *L. monocytogenes* in a single serving once every 2 years without apparent subsequent disease [10]. Likewise, it has been estimated that each human individual in Western countries is exposed to *L. monocytogenes* multiple times per year. Moreover, 5%–10% of healthy asymptomatic humans may be gastrointestinal carriers of *L. monocytogenes* [23]. These observations have suggested that in most cases, human exposure to *L. monocytogenes* does not lead to disease [23].

Hospitals can also be a significant source of listerial infection because of the combination of access to RTE foods and a higher probability of immunosuppressed consumers [2, 21]. Thus, surveillance strategies involving highly complex epidemiological, microbiological, and molecular methods are necessary for healthcare facilities to track the sources of nosocomial *L. monocytogenes* infection. These methods have validated several vehicles of foodborne listerial disease among inpatients. Hospital kitchenware [24], hospital milkshake makers [25–27], production environment of food supply companies [25, 28–34], pasteurized dairy-based ice cream products [25], meat jelly [30], RTE meat products [28], prepacked sandwiches [20, 32, 35], butter [20, 34], precut celery [20, 36], Camembert cheese [20, 29], sausage [20, 33], tuna salad [20, 37], chicken salad [36], and profiteroles [31] served to inpatients have been identified as sources of infection in nosocomial cases and outbreaks of invasive listeriosis. Using molecular biology and microbiological methods, these aforementioned sources of infection have tested positive to the same sequence type or the same serotype of *L. monocytogenes* strains isolated from patients with hospital-acquired neurolisteriosis and/or listerial septicemia reported in more than 15 countries from North America, Europe, Asia, and Oceania [20, 24–37].

In developed countries, policies exist to ensure food safety and prevent community listerial infection. According to European regulations, *L. monocytogenes* levels of ≤ 100 CFU/g are allowed in RTE foods within shelf life as a food safety criterion [38], whereas the standard for the US Food and Drug Administration (FDA) is no detectable *L. monocytogenes* per 25 g sample of RTE food [20]. However, some investigations have found levels below these thresholds in finished food products associated with outbreak cases of invasive listeriosis [32, 34]. Other control measures to reduce the incidence of community-acquired listerial infections include monitoring and sanitation of food production plants, and educational programs targeted to consumers and risk groups [1, 2]. Likewise, to minimize the risk of foodborne listeriosis among inpatients, recommendations addressed to hospital kitchens and hospital food suppliers have been published [37, 39–42]. These recommendations are intended to implement food preparation policies, routine and thorough cleaning of food contact surfaces in hospital kitchens and production facilities, and avoid serving high-risk foods to patients at risk for listeriosis [37, 39–42].

## 4. Microbiology

*Listeria monocytogenes* is a facultative anaerobic, Gram-positive rod, which grows well on blood agar, producing an incomplete zone of β-hemolysis [1, 10, 43–45]. Although optimal growth occurs at 30°C to 37°C, *L. monocytogenes* may be able to grow at temperatures as low as 4°C [44, 46]. Routine media are effective for isolating *L. monocytogenes* from samples of normally sterile sites (CSF, blood) [44]. During the Gram staining process, the organisms may appear as Gram-variable or Gram-negative rods that could lead to laboratory misidentification [1, 44, 45, 47].

*L. monocytogenes* is one of seven species of *Listeria* and the most commonly isolated organism in the genus [48]. There are more than 14 serotypes of *L. monocytogenes*; four of these serotypes (1/2a, 1/2b, 1/2c, and 4b) are involved in more than 95% of reported human listeriosis cases and outbreaks [8, 21, 44, 48]. Serotype 4b is most often responsible for listerial meningoencephalitis in more than 80% of cases [5, 8, 21]. Serotypes 1/2a, 1/2b, and 4b have also been reported from outbreak cases of hospital-acquired invasive listeriosis (meningitis and/or bacteremia) [20, 32, 33].

## 5. Pathogenesis

### 5.1. Virulence Factors

Pathogenesis of *L*. *monocytogenes* could be divided into two virtual stages, a cell invasion and an intracellular life cycle, where several virulence factors implicated may also be involved in CNS tropism. In the human host, following ingestion of contaminated food, *L. monocytogenes* can enter through the intestinal mucosa by invading nonphagocytic cells, such as epithelial cells [6, 49]. *L. monocytogenes* invasion is mediated by two members of a bacterial surface protein family, named internalin; internalin A (InlA) and internalin B (InlB) interact with their specific host cell receptors, E-cadherin and Met, respectively [11, 50, 51]. E-cadherin is a cell surface receptor located at the intestinal barrier, the BBB, and the placenta, and Met is a ubiquitous cell surface receptor [50]. This interaction triggers listerial engulfment into the host cell membrane and formation of an internalization vacuole [50, 52, 53], leading to the intracellular life cycle of *L. monocytogenes*.

Once internalized in the host vacuole, *L. monocytogenes* secretes three more virulence factors, the pore-forming cytotoxin listeriolysin O (LLO), the phosphatidylcholine-specific phospholipase (PC-PLC), and the phosphatidylinositol-specific phospholipase C (PI-PLC) [54]. These virulence factors are responsible for the rupture of the single vacuolar membrane, which releases the bacterium into the host cell cytoplasm [54]. In the cytoplasm, *L. monocytogenes* replicates and produces the bacterial surface actin assembly-inducing (ActA) protein that recruits the actin-related protein (Arp2/3) complex to initiate polymerization of host cellular actin monomers into an actin filament network at one pole of the bacterium. Thereby, the actin polymerization creates a comet tail leading to a propulsive force that propels the bacterium through the cytoplasm back toward the host cell surface to invade adjacent cells [1, 6, 53–55]. The efficient cell-to-cell spread of motile *L. monocytogenes* requires the interaction of the bacterial surface protein internalin C (InlC) with a host adapter protein named Tuba; this interaction perturbs cortical membrane tension of the infected cell and favors *L. monocytogenes* protrusion formation [2, 52, 53, 56]. When bacteria leave a host epithelial cell to invade another, they are endocyted by the adjacent cell, entrapped within the double membrane (one from the cell that has just left and one from the newly infected vacuole), and released into the adjacent cell cytoplasm by the combined action of LLO and phospholipases. Thus, *L. monocytogenes* spreads safely through host tissues without leaving the host cytosol and is protected from the host-adaptive immune system [6, 57].

### 5.2. CNS Invasion

After crossing the intestinal epithelial barrier, *L. monocytogenes* can reach the CNS by hematogenous dissemination [11, 57, 58]. *L. monocytogenes* survives and proliferates not only in nonphagocytic cells but also in phagocytic cells such as neutrophils, monocytes, macrophages, or dendritic cells, indicating that *L. monocytogenes* may reach the BBB through these infected cells [1, 11]. As mentioned above, the BBB is also composed of cells that express E-cadherin and Met, such as the cerebral capillary endothelium and choroid plexus epithelium. These receptors also enable *L. monocytogenes* to cross the BBB via expression of InlA and InlB, similar to when *L. monocytogenes* crosses the intestinal barrier. Furthermore, LLO secreted by *L. monocytogenes* also induces NFkB activation, both in endothelial cells and in brain microvessels. This motivates the expression of the surface adhesion molecules P-selectin and ICAM-1, which allow infected cells to adhere to brain microvessels or meningeal vessels, favoring CNS invasion [11] (Table 1).

As mentioned, when *L. monocytogenes* is inside nonphagocytic cells, it evades the innate and humoral immune response and moves from cell to cell without contact with the extracellular milieu. However, once *L. monocytogenes* is engulfed by a phagocyte, it is killed within the phagosome. Thus, resistance to infection with *L. monocytogenes* depends primarily on cell-mediated immunity, and when this cell-mediated immunity is defective, the susceptibility to infection with *L. monocytogenes* is increased [11, 59, 60].

Further mechanisms of infection have been described for listerial rhombencephalitis and listerial CNS abscess. Animal model studies have suggested that *L. monocytogenes* can also gain access to the CNS via a neural retrograde route [1, 11, 49]. It means that *L. monocytogenes*, via the oral route, could cross the oral epithelium and reach the CNS through retrograde axonal migration along cranial nerves [1]. This pathway would be used by *L. monocytogenes* to induce rhombencephalitis [11, 49]. In this sense, radiological findings by magnetic resonance imaging (MRI) from listerial rhombencephalitis cases have shown early involvement of the trigeminal nerve with spread into the cerebellopontine angle and further along the trigeminal sensory tracts into the pons and medulla oblongata [61]. Likewise, a review of 120 patients with listerial rhombencephalitis showed that the cranial nerves innervating the oropharynx (VII, V, IX, and X), medulla oblongata, cerebellum, and pons were the most frequently involved brain structures by clinical and imaging assessment, respectively [61]. However, the molecular mechanisms of this proposed pathway have not been well elucidated [11]. Whereas, the hematogenous route of infection has also been described for listerial CNS abscess, in which *L. monocytogenes* may reach the brain parenchyma via the cerebral capillary endothelium. Thus, it has been suggested that *L. monocytogenes*-infected macrophages may pass through endothelial cells via the middle cerebral artery resulting in cerebritis, which leads to brain abscess formation [58, 60, 62].

## 6. Clinical Features

Listeriosis can manifest as an invasive or noninvasive disease [21]. In adults, invasive listeriosis is more common among immunocompromised people and can lead to neurolisteriosis, septicemia, or maternal–neonatal infection [1, 2, 12, 18, 21, 63].

Seeking a diagnosis of neurolisteriosis is primarily encouraged due to the presence of its risk factors, as the clinical findings of neurolisteriosis could resemble those of any other community-acquired CNS infection caused by more common pathogens [64]. Nevertheless, the ability of *L. monocytogenes* to cause meningoencephalitis more than isolated meningitis could make the difference from other bacteria more frequently associated with meningeal signs than with brain parenchymal signs, such as *S. pneumoniae*, *N. meningitidis*, and *H. influenzae*.

Risk factors for neurolisteriosis in order of frequency include age ≥ 50 years, any corticosteroid/immunosuppressive therapy, solid organ cancer, hematological malignancy, diabetes mellitus, chronic liver disease, alcoholism, chronic kidney disease (CKD), inflammatory rheumatic disorders, inflammatory bowel diseases, and HIV infection [8, 19, 59, 64–71]. At least one of these risk factors is present in more than 85% of cases, and patients with a prior history of receiving immunosuppressive therapy within 1 month and malignancy are the most significant predictive risk factors for neurolisteriosis [59, 65, 66, 68, 69, 71]. Whereas, only a low proportion (4%) of adults with neurolisteriosis are younger than 50 years and immunocompetent [66, 68]. Neurolisteriosis can manifest as meningoencephalitis in roughly 85% of cases, or meningitis without encephalitis in 13%, clinical rhombencephalitis in 9%–20%, and brain abscesses in 1%–10% of cases [5, 58, 59, 65, 68, 70]. Similar to common causes of meningitis, the clinical presentation of listerial meningoencephalitis may have an acute or subacute course, with a duration of symptoms before admission of 2–5 days in most cases; but up to 50% of patients have shown symptoms for more than 5 days before hospitalization [8, 59, 65, 67].

Most patients with neurolisteriosis show altered mental status (50%–97%), with a mean score on the Glasgow Coma Scale (GCS) of 12, and a GCS of < 8 (coma) in 10%–26% of cases [7, 59, 66–68, 71]. Likewise, fever (85%–100%), headache (46%–98%), and neck stiffness (61%–75%) are common presentations [8, 59, 65–68]. Other manifestations more associated with clinical encephalitis than with meningitis in neurolisteriosis are epileptic seizures reported in 3%–31%, focal neurological signs (hemiparesis, aphasia, and dysarthria) in 13%–35%, cerebellar syndrome in 4%–39%, nystagmus in 28%, at least one cranial nerve paralysis (particularly V, VI, VII, IX, or X, and less commonly II, III, IV, VIII, XI, or XII) in 17%–45%, and multiple cranial nerve paralysis in 5% of cases [7, 8, 59, 61, 65–68, 70, 71].

The classic triad of meningitis (fever, neck stiffness, and change in mental status) is present in 33%–49% of cases with listerial meningitis [7, 8, 64, 66, 67]. This is similar to what is observed for other bacterial meningitis (39%) [8]. Likewise, a lack of meningeal signs has been described in 8%–42% of cases with neurolisteriosis [59, 64, 65]. This contrasts with the lack of meningeal signs reported in roughly 10% of meningitis cases due to other common pathogens [59]. An immunocompromised state has been associated with a lack of meningeal signs in neurolisteriois [8].

Listerial rhombencephalitis or brainstem encephalitis is a type of neurolisteriosis that affects the pons, mesencephalon, medulla oblongata, or cerebellum [65, 71, 72]. Listerial rhombencephalitis occurs predominantly in middle-aged adults, without any predisposing condition in 70%–80% of cases, and is characterized by acute and progressive brainstem dysfunction [60, 61, 64, 65]. Clinical presentation of listerial rhombencephalitis classically appears in two phases. In the first 4–10-day period, headache, malaise, nausea or vomiting, and fever can be present. In a subsequent period, single or multiple asymmetrical cranial nerve deficits (mainly VII, VI, V, IX, and X, in order of frequency), cerebellar signs (ataxia, ataxic gait, vertigo, and cerebellar dysarthria), and hemiparesis appear, but less than half exhibit meningeal symptoms (44%–46%) [61, 64, 71, 72]. Rhombencephalitis has been typically associated with neurolisteriosis. Furthermore, *L. monocytogenes* is the most common cause of infectious rhombencephalitis; nevertheless, tuberculosis, *Brucella* spp., *Human herpes* virus 6, *Herpes simplex* virus, Enterovirus 71, *Epstein–Barr* virus, Behçet's disease, and other infectious, autoimmune, and paraneoplastic syndromes might cause brainstem involvement as well [65, 71, 72].

Clinical presentation of listerial brain abscess is characterized by fever in roughly 75% of cases, gradual onset focal neurological deficits (hemiparesis, limb weakness, sixth, and/or seventh cranial nerve palsy) in 75%–87% of patients, abnormal sensorium and headache in roughly 40% each, and meningeal signs may be absent in 75% of cases [1, 59]. In most patients, these symptoms secondary to listerial brain abscess may be present for 1-2 weeks at admission [1]. Listerial brain abscesses have also been reported in people ≥ 50 years (median, 55 years) and with at least one predisposing condition for neurolisteriosis in more than 80% of cases [45, 48, 58, 59]. In adults with immunosuppression, the differential diagnosis of listerial abscess(es) could include other pyogenic brain abscesses, CNS lymphoma, toxoplasmosis, nocardiosis, tuberculosis, cryptococcosis, and aspergillosis [45].

Most adults with nosocomial bacterial meningitis have a history of neurosurgery, otorhinolaryngologic surgery, skull fracture, neurosurgical device, CSF leak, altered immune state, or a distant focus of infection [64, 73]. Etiology in these cases mainly includes aerobic Gram-negative bacilli, *S. pneumoniae*, staphylococci, or streptococci [64, 73]. None of these underlying conditions have been described when nosocomial neuroinfection is caused by *L. monocytogenes*, except for the altered immune state [73]. The majority of nosocomial neurolisteriosis cases are also immunocompromised adults with cancer (solid tumor or hematologic malignancy), on immunosuppressive therapies (corticosteroids or cytotoxic medications), or with predisposing conditions (diabetes mellitus, blood disorder, pregnancy, cardiovascular, renal, pulmonary, liver, or autoimmune diseases) [18–20, 24, 28–30]. Two case series showed that 80%–100% of adults with hospital-acquired neurolisteriosis were immunocompromised [20, 59, 74].

Clinical presentations of hospital-acquired neurolisteriosis cases reported mainly include meningitis and meningoencephalitis [20, 28–30, 74, 75], and less commonly multiple abscesses in the cerebrum and/or cerebellum [45]. In a patient who develops neurolisteriosis during a hospital stay, the wide range of the incubation period for this disease makes it difficult to distinguish between a community-acquired and a hospital-acquired case. The overall median incubation period for invasive listeriosis is 10 days, ranging from 0 to 70 days depending on the type of listerial disease (median incubation period of 10 days, ranging from 0 to 21 days for CNS cases) [21, 63]. Most of the hospital-acquired neurolisteriosis cases described in the literature have their source of infection identified on RTE-foods served to inpatients [20]. Nevertheless, some authors have only considered the time of symptoms onset after admission to, or after discharge from, the hospital to define nosocomial cases [19, 59, 74]. In a nosocomial listeriosis outbreak, the incubation period of two neurolisteriosis cases was documented to be 4 days and ≤ 24 days, respectively [29]. Camembert cheese was the source of this outbreak [29]. In another nosocomial outbreak of invasive listeriosis, involving fifteen patients with neurolisteriosis and/or listerial bacteremia, symptoms onset ranged from three to 140 days after hospital admission, with a mean of 25 days. Raw vegetables were suggested as the sources of infection [75]. And authors from two retrospective studies with four and three cases of hospital-acquired neurolisteriosis, respectively, found that symptoms onset ranged from 7 to 44 days after admission, but without clarifying the sources of these infections [19, 74] (Table 2).

## 7. Neuroimaging Findings

Patients with neurolisteriosis show abnormal images on MRI or computed tomography (CT) in 83% of culture-proven cases [70]. Nevertheless, MRI is more accurate than CT in detecting typical CNS lesions caused by *L. monocytogenes*. Therefore, neuroradiological findings have been reported normal on cranial MRI in 4%–37% of cases, and on cranial CT scan in 23%–70% of cases with neurolisteriosis [65, 66, 70].

Nonspecific supratentorial white matter lesions described in 59%–99% of cases [70, 71], and meningeal enhancement reported in 35%–63% of cases [65, 70], are the most common patterns of radiological neurolisteriosis on MRI and CT. Brainstem involvement and brain abscesses are less common findings. Brainstem radiological involvement may be seen in up to 67% of cases among patients with listerial encephalitis evaluated only by MRI [71], but this radiological lesion can be detected in as few as 10% of neurolisteriosis cases evaluated on both CT and MRI [65, 70]. Whereas, abscesses and/or nodular lesions suggestive of brain abscesses have been observed in 4%–15% of neurolisteriosis cases on CT and MRI [66, 70], and in 56% of cases among patients with listerial encephalitis by MRI only [71]. Most of the listerial brain abscesses are solitary, and multiple abscesses occur in roughly a quarter of cases [1, 60, 76]. These lesions are observed by neuroimaging at the infratentorial (brainstem) level in 50%, at the supratentorial level in 40%, and both in 10% of cases [62, 70].

Other neuroradiological findings in neurolisteriosis are dilated Virchow–Robin spaces (31%), hydrocephalus (2%–19%), parenchymal hemorrhages (15%), ischemia (11%), radiological vasculitis (5%), and contrast-enhancing ventricles (3%) [65, 66, 70, 71, 77, 78]. Lesions reported on cranial MRI (mainly T2 hyperintensity, contrast enhancement, abscess, focal bleeding, and ischemia) may also be located at the cerebellum and basal ganglia in ≤ 20%, and at the internal capsule, thalami, spinal cord, supratentorial cortex, and periaqueductal gray matter in less than 10% of cases [70, 71].

Although MRI is superior to CT for detecting CNS lesions in neurolisteriosis, a CT scan is often necessary first. Cranial CT has been recommended before lumbar puncture in patients with suspected bacterial meningitis, mainly in those cases presenting with new-onset seizures, focal neurological deficits, a moderate-to-severe impairment of consciousness, or an immunocompromised state [79–81]. Any of these clinical criteria suggests a brain shift, and cranial CT must be performed before lumbar puncture in these cases as a precaution to exclude brain shift and reduce the risk of brain herniation due to the withdrawal of CSF through the lumbar puncture [79, 81]. The well-known advantages of CT scan (faster and less sensitive to patient movement during examination than MRI) would allow timely information to be displayed for this purpose. Of note, since neuroimaging has been identified as a risk factor for delaying antibiotic treatment, it has also been recommended to start antimicrobial therapy before performing cranial CT [79–81]. Furthermore, given that an initial cranial CT may be normal in many patients with neurolisteriosis [65, 66, 70], those cases with clinical rhombencephalitis but a cranial CT without characteristic lesions must undergo MRI that is superior in detecting subtentorial lesions, including brainstem lesions [1, 44, 65, 71, 82]. However, brainstem radiological involvement may not always correlate with clinical rhombencephalitis [70]. It has been reported that only 57% of neurolisteriosis patients with brainstem radiological involvement exhibit brainstem symptoms and that two-thirds of patients exhibiting clinical brainstem signs do not have radiological brainstem lesions on MRI or CT scan [70].

**Figure 1:**
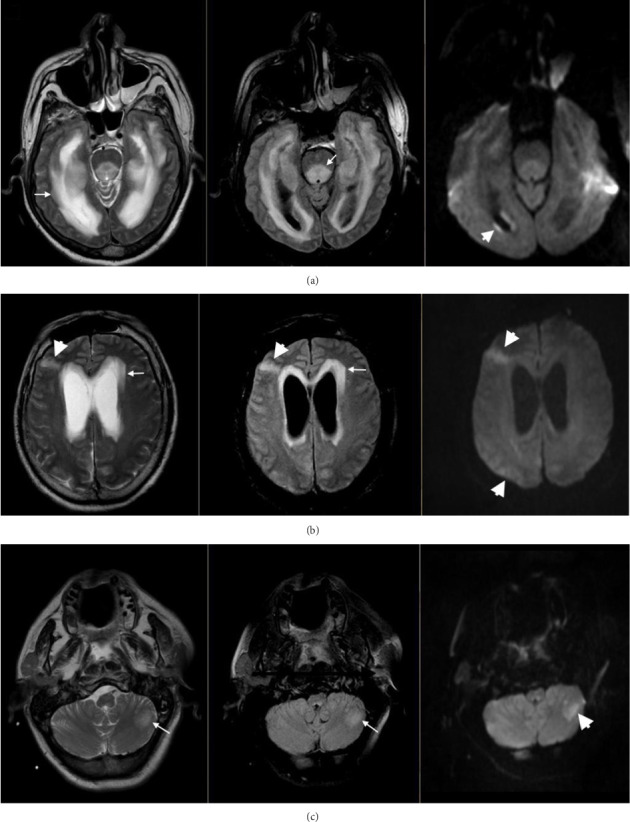
MRI findings of nosocomial neurolisteriosis in a 61-year old man with non-Hodgkin's lymphoma on chronic corticosteroid therapy. On Day 45 of hospital stay for cancer treatment, he presented with meningoencephalic symptoms. MRI was performed on Day 3 after neurological symptoms onset. From left to right, axial T2-weighted, FLAIR, and DWI sequences. (a) Diffuse hyperintense signal of the periventricular white matter and the periaqueductal region of the mesencephalon (arrows) and focal lesions of the right side periventricular white matter with increased DWI evocative of acute ischemic images (arrow head). (b) Hydrocephalus with periventricular transependymal edema (arrows), and focal lesions with hyperintense signal of the right frontal and parietal cortical matter on T2-weighted and FLAIR sequences with increased DWI evocative of acute ischemic images (arrow heads). (c). Focal left cerebellar lesion hyperintense on T2-weighted and FLAIR sequences (arrow) with increased DWI evocative of acute ischemic image (arrow head). FLAIR = flow attenuated inversion recovery. DWI = diffusion weighted imaging.

Descriptions of neuroradiological images in hospital-acquired neurolisteriosis are scarce. Nevertheless, in our experience, we recorded one patient with nosocomial neurolisteriosis from our hospital in Ecuador, whose brain MRI showed a combination of multiple parenchymal and meningeal lesions (Figure 1).

## 8. Laboratory Diagnosis

### 8.1. CSF Analysis

As in all cases of CNS infection, CSF examination is mandatory. In comparison with bacterial meningitis due to other common pathogens (*S. pneumoniae*, *N. meningitidis*, *H. influenzae*, *Escherichia coli*, and *Streptococcus agalactiae*), the CSF profile in most patients with listerial meningitis/meningoencephalitis has shown significantly lower pleocytosis, lower percentage of neutrophils, lower protein concentrations, and less hypoglycorrhachia [8, 59, 67, 69]. Similar to listerial meningitis/meningoencephalitis, the CSF profile of listerial rhombencephalitis has shown a low-grade pleocytosis, with about an equal percentage of mononuclear and polymorphonuclear cells, mild hyperproteinorrachia, and a hypoglycorrhachia found in only 21% of cases [64, 72]. Likewise, CSF findings in neurolisteriosis with focal lesions (brain abscess/cerebritis) have shown no pleocytosis in 30% of cases, hyperproteinorrachia with a median of 76 mg/dL, and hypoglycorrhachia in only 20% of patients [59]. Even those cases with confirmed listerial meningitis often exhibit a lack of biological signs of bacterial meningitis [67]. In a cohort study of 30 patients with confirmed listerial meningitis by culture of CSF, 23% of them were reported with no individual CSF findings indicative of bacterial meningitis [67]. This lack of CSF findings is more frequent in neurolisteriosis compared to other causes of bacterial meningitis [64]. In a cohort study of 258 adults with culture-proven meningococcal meningitis, CSF examination was normal in 1.7% of these patients [64].

**Figure 2:**
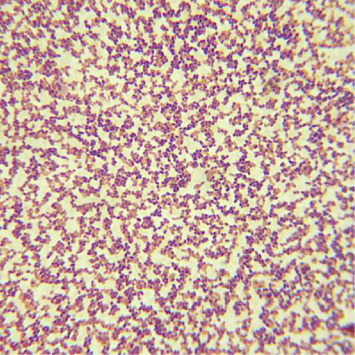
Neurolisteriosis in a 61-year-old man with non-Hodgkin's lymphoma on corticosteroid therapy (from the case of Figure 1). Gram stain from growth of CSF culture shows Gram-variable short rods, magnification × 100. Note interspersed Gram-negative and Gram-positive short rods.

Gram-positive rods are revealed on CSF Gram stain in only 6%–41% of patients with neurolisteriosis [5, 7, 8, 59, 64–69, 78], and this yield is even lower for patients with listerial rhombencephalitis (14%) and listerial brain abscess (< 4%) [62, 64]. The yield of CSF Gram stain for neurolisteriosis is also lower than that reported for pneumococcal (69%–93%) and meningococcal (30%–89%) meningitis [59, 64, 69]. Furthermore, *L. monocytogenes* on CSF Gram stain can be mistaken for another organism in many cases [59, 73]. In different growth media, *L. monocytogenes* may appear as short rods, longer rods, or elliptical cocci on Gram stain [44]. In addition, *L. monocytogenes* tends to be over-decolorized by alcohol during the Gram staining process and may appear as Gram-negative or Gram-variable [1, 44, 45, 59]. Gram variability is described as a proportion of nonviable Gram-negative cells interspersed throughout the entire population of Gram-positive cells (Figure 2), observed on Gram stain of cultures [83].

### 8.2. CSF and Blood Cultures

The yield of CSF cultures is optimal in listerial meningoencephalitis, whereas blood cultures could be more useful in listerial rhombencephalitis and brain abscesses [59]. Among all clinical presentations of neurolisteriosis, positive CSF cultures have been found in 56%–96% of cases [5, 8, 65, 66, 68, 69, 78], and positive blood cultures in 16%–75% of cases [5, 7, 8, 59, 65–69, 78]. Furthermore, patients with confirmed neurolisteriosis have shown *L. monocytogenes* isolated from both CSF and blood samples in roughly 25%–74% of cases [7, 18, 68, 69]. In contrast, the yield of CSF culture is lower than blood culture when neurolisteriosis is more restricted to parenchymal involvement, as with listerial rhombencephalitis and brain abscesses. In patients with listerial brain abscess, positive CSF cultures have been reported in 20%–51% of cases, and positive blood cultures in roughly 80%–86% of cases [1, 58, 59]. In addition, brain abscess cultures have also been reported to have a low yield [58]. Thus, listerial brain abscess biopsy material can be positive in only 50% of cases [58]. Likewise, in patients with listerial rhombencephalitis, positive CSF cultures have been found in 33%–41% of cases, and positive blood cultures in 46%–79% of cases [1, 71, 72]. Therefore, blood samples for cultures must also be collected early for best diagnostic performance in all cases, especially in those patients with brain parenchymal lesions and lack of meningeal involvement, whose CSF cultures could be nondiagnostic.

## 9. Treatment

### 9.1. Antimicrobial Therapy

According to IDSA and ESCMID guidelines, empirical antimicrobial therapy for C-ABM in adults must comprise vancomycin plus a third-generation cephalosporin (ceftriaxone or cefotaxime) [79, 84]. For patients aged 50 years or older, particularly in those with immunosuppression, an aminopenicillin or penicillin G should also be included for additional coverage of *L. monocytogenes* [64, 65, 79, 84].

An aminopenicillin (ampicillin 2 g IV every 4 h, or amoxicillin 2 g IV every 4–6 h) remains the drug of choice for neurolisteriosis treatment, and it is the antibiotic associated with the highest favorable outcomes [19, 68, 85]. Penicillin G (four million units IV every 4 h) has been described with comparable efficacy to aminopenicillins for neurolisteriosis [22, 84, 85]. No other antibiotic is recommended as first-line therapy for neurolisteriosis in adults.

For those cases unresponsive to first-line therapy, or with β-lactam allergy, TMP-SMX (10–20 mg/kg IV every 6–12 h, dosage based on trimethoprim component) as monotherapy is the second choice therapy of listerial CNS infection [59, 72, 79, 84, 86]. *L. monocytogenes* susceptibility to this antibiotic is high [87]. Although TMP-SMX has not been demonstrated to be as effective as aminopenicillins in neurolisteriosis [68, 87], it shows better outcomes than other antimicrobials when penicillin or aminopenicillin administration is contraindicated in neurolisteriosis [68].

Practice guidelines recommend adding aminoglycosides (gentamicin) to aminopenicillin or penicillin treatment [79, 84]. Some in vitro and in vivo studies have shown a synergistic and bactericidal effect of this combination therapy, but gentamicin has no activity against intracellular bacteria in vivo [22, 59, 85, 87]. It also has a limited capacity to cross the BBB [17, 87], and its well-known nephrotoxicity limits the daily dose for a short period. Even so, a cohort study suggested that amoxicillin/gentamicin combination therapy was associated with improved survival in comparison to amoxicillin alone for patients with invasive listeriosis (neurolisteriosis and bacteremia), but without establishing a separate analysis of these two listerial invasive diseases [68]. Another retrospective review that included 284 patients with neurolisteriosis showed a lower mortality rate when ampicillin or penicillin was combined with an aminoglycoside (mortality rate, 14%) compared to ampicillin or penicillin as monotherapy (mortality rate, 22%) [59]. The authors in this review suggested adding an aminoglycoside to penicillin or ampicillin only during the first 7–10 days of therapy for uncomplicated cases [59]. Many other studies, most of them retrospective, suggest a deleterious or not statistically significant effect from adding aminoglycoside to treat neurolisteriosis in comparison to aminopenicillin in monotherapy [5, 8, 17, 65, 66, 71, 85]. Therefore, adding an aminoglycoside to β-lactam for neurolisteriosis therapy may be considered, but treating physicians should also be cautious, especially in terms of renal failure [5, 84].

Another combination therapy not included in the guidelines was tested in a retrospective study published 30 years ago [87]. It showed that the combination therapy of aminopenicillin (ampicillin or amoxicillin) with cotrimoxazole was better than aminopenicillin with or without aminoglycoside for treating listerial meningoencephalitis [87]. In those cases with consciousness disturbances, general seizures, and/or focal neurological signs, this combination therapy demonstrated lower treatment failure (6.7% vs. 57%), lower mortality related to *L. monocytogenes* (6.7% vs. 23·5%), and lower morbidity (13.3% vs. 60%) compared to aminopenicillin with or without aminoglycoside [87]. Authors argued that the adjunction of the bactericidal effect of cotrimoxazole to the bacteriostatic effect of an aminopenicillin, and the good CNS penetration of both drugs could explain their results [86, 87]. Nevertheless, the number of cases included in this study was small (22 cases), and no further prospective studies with this combination therapy have been carried out [87].

Alternative therapies are required in those patients who develop drug allergy or toxicity to both aminopenicillin (or penicillin) and TMP-SMX, or in those cases that appear to be not responsive to these antibiotics. For these situations, there is limited experience in the treatment of neurolisteriosis, based on case reports. Such is the case with linezolid, which has been used in combination therapy for patients with listerial rhombencephalitis and brain abscess [88–90]. In anecdotal cases, initial antibiotics were shifted to linezolid in combination with rifampin, meropenem, or TMP-SMX after allergic reaction, toxicity, or failure associated with standard therapy [88–90]. Experience in these cases showed clinical, neuroradiological, and CSF profile improvement after weeks or months of linezolid in combination therapy [88–90]. In this regard, optimal antimicrobial activity in vitro against *L. monocytogenes*, adequate CSF penetration, and efficacy demonstrated in animal models with listerial CNS infection have been argued to use linezolid for neurolisteriosis [88–90]. However, an important concern is linezolid-associated bone marrow suppression, when it is used for prolonged periods [89].

As with TMP-SMX and linezolid, moxifloxacin is also cited in guidelines as an alternative therapy for neurolisteriosis [79]. According to in vitro and animal models, moxifloxacin can reach high concentrations in the CNS [91–93]. And, compared to aminopenicillins with or without gentamicin, moxifloxacin achieves similar bactericidal activity against *L. monocytogenes* in the intra- and extracellular milieu [22, 91, 92, 94, 95]. However, in cells infected with *L. monocytogenes*, the activity of moxifloxacin against the intracellular form is 5- to 20-fold weaker than that against the extracellular form of this organism [22, 96–98]. And as described, *L. monocytogenes* is a facultative intracellular bacterium; therefore, the antibiotic efficacy is dependent on the ability to eliminate the intracellular reservoirs of bacteria [95]. Furthermore, neurolisteriosis case reports or clinical trials using moxifloxacin are needed to show its clinical potential in this disease [91].

Meropenem, vancomycin, and third-generation cephalosporin, which are mostly used as empirical therapy for acute bacterial meningitis, report failure after treatment as monotherapy in neurolisteriosis. Although meropenem shows a minimum inhibitory concentration comparable to ampicillin against *L. monocytogenes*, this carbapenem class antibiotic has demonstrated clinical failure and higher mortality compared to receiving aminopenicillins or benzylpenicillin in adults with neurolisteriosis, probably because of differences in extracellular and intracellular antibiotic activity between both antibiotic classes [22, 85]. A retrospective study reported a higher mortality rate in cases with invasive listeriosis (bacteremia/meningitis or both) receiving definitive therapy with meropenem compared with those who received benzylpenicillin or aminopenicillins [85]. Vancomycin has demonstrated good activity against *L. monocytogenes* in vitro [99]; nevertheless, clinical experience is sparse and treatment failure has been reported in neurolisteriosis cases [59, 100, 101]. No currently available cephalosporin is recommended for neurolisteriosis, even third-generation cephalosporin, due to a documented high prevalence of resistant strains of *L. monocytogenes* to this antibiotic class [99]. Other antimicrobials with in vitro activity against *L. monocytogenes* include rifampin, imipenem/cilastatin, chloramphenicol, tetracycline, and erythromycin. But antimicrobial resistance (AMR) and high rates of treatment failures with these antibiotics in monotherapy or combination therapy have also been described [22, 59, 88].

*L. monocytogenes* is intrinsically resistant to several antibiotics (cefuroxime, cefotaxime, ceftriaxone, cefepime, aztreonam, oxacillin, clindamycin, nalidixic acid, fosfomycin, fusidic acid, and sulfonamides), some of which are recommended as empirical therapy for C-ABM in adults [79–81, 84, 102–105]. Nevertheless, antimicrobial susceptibility testing of recommended antibiotics for neurolisteriosis shows a good activity profile, which remains over time. According to the recent regional studies from European countries (France, Italy, and Russia), AMR to first-line antibiotics (aminopenicillins, penicillin G, and aminoglycosides) is almost absent for *L. monocytogenes* strains from clinical samples [102, 103, 105]. Likewise, it has been reported that the occurrence of strains resistant to second-line antibiotics for neurolisteriosis (TMP-SMX, linezolid, and moxifloxacin) is low and it does not exceed 5% [102, 103], but this AMR pattern may differ in other regions. For instance, in an Italian study, 29% of *L. monocytogenes* isolates from clinical samples were resistant to TMP-SMX [105]. In contrast, a worldwide study suggested that the antibiotic resistance rate is increasing for first-line therapy of listeriosis [104]. This meta-analysis of 33 studies (most of them from developed countries) showed that 10%, 6%, 19%, and 6% of *L. monocytogenes* strains from human sources were resistant to ampicillin/amoxicillin, gentamicin, penicillin, and TMP-SMX, respectively [104]. However, the authors addressed limitations because data were collected from a few countries, heterogeneity among included studies was considerable, and there was an unclear methodology for antibiotic susceptibility testing in some included studies [104]. Hence, the current antibiotic options for neurolisteriosis remain microbiologically effective according to some studies made in developed countries [79, 84, 102, 103, 105]. Nonetheless, an accurate global surveillance system is needed to monitor the trend of AMR in *L. monocytogenes* that may vary in different regions worldwide [103–105].

Guidelines recommend ≥ 21 days of antimicrobial therapy for neurolisteriosis, emphasizing that the duration of therapy may be individualized based on the patient's clinical/radiological response [79, 84]. In addition, the clinical presentation of neurolisteriosis should also be considered to decide the duration of antimicrobial therapy. According to retrospective studies, surviving patients with listerial meningoencephalitis have been treated for a median of 3 weeks (range, 1–90 days) [59], whereas surviving patients with listerial rhombencephalitis or brain abscesses have been treated for at least 6–8 weeks or longer [58, 59, 65]. Furthermore, to estimate the duration of antimicrobial therapy for listerial rhombencephalitis and brain abscess, a follow-up by serial neuroimaging may be required until the lesions are resolved [45, 58, 59, 65].

### 9.2. Adjunctive Therapy

Adjunctive use of dexamethasone is recommended in adults with C-ABM based on a lower percentage of hearing loss, neurologic sequelae, and mortality when *S. pneumonia* or *H. influenzae* are the suspected or proven meningeal pathogens [79, 84]. But if *L. monocytogenes* is identified, it has been suggested that dexamethasone be withdrawn due to its potential detrimental outcomes [80, 106]. This advice derives from the results of a French cohort study, which found a higher mortality of 47% in patients with neurolisteriosis who received adjunctive dexamethasone versus 27% mortality in those cases who did not receive dexamethasone [68]. However, in this study, only the minority of patients (13% of 252 neurolisteriosis cases) received adjunctive dexamethasone [68]. And it was not specified whether these patients were prescribed adjunctive dexamethasone according to guideline recommendations or according to a standard protocol. Nevertheless, the authors stated that dexamethasone was given within 24 h of the first antibiotic dose [68, 79, 84]. Conversely, a more recent Dutch cohort study found that adjunctive dexamethasone was associated with improved outcomes in patients with *L. monocytogenes* meningitis [66]. In this cohort study, 52% of 161 patients with neurolisteriosis were treated with adjunctive dexamethasone according to guideline recommendations (10 mg QID for 4 days, starting with the first dose of antimicrobial therapy or within 4 h after initiation of antibiotic treatment) [66, 79]. The rate of unfavorable outcomes and death was higher in patients who did not receive adjunctive dexamethasone (72%), as compared to those who received adjunctive dexamethasone (46%) [66]. And this rate was also higher in those cases who did not complete the full course of 4 days with adjunctive dexamethasone (60%) and in those who received a different regimen of dexamethasone (55%) [66]. At least three other earlier studies have also assessed the role of adjunctive dexamethasone in adults with neurolisteriosis [5, 7, 8]. These cohort studies found that adjunctive dexamethasone was associated with lower neurological sequelae, a trend toward higher survival, or no harm in neurolisteriosis [5, 7, 8]. In one of these three studies, the dose of adjunctive dexamethasone was not in concordance with guidelines recommendations [5, 79, 84], and in another study, the dose and duration of dexamethasone treatment were not specified [8]. However, the Dutch study and these three earlier studies have in common the timing of dexamethasone treatment, which was started minutes before or concomitantly to the first antibiotic dose [5, 7, 8, 66], whereas in the French study, there was a lack of information about the specific dose, duration, and timing of dexamethasone (other than within 24 h of first antibiotic dose) [68]. Thus, it is not clear if, in the French study, dexamethasone was started when it had ceased to be beneficial. Early initiation of dexamethasone treatment for acute meningitis (minutes before, or up to 4 h after the first antibiotic dose) has been recommended to reduce the inflammatory response from rapid bacteriolysis by antibiotic therapy [66, 79]. The studies that started dexamethasone at the recommended time showed that adjunctive dexamethasone was not associated with harm or improved outcomes in neurolisteriosis [5, 7, 8, 66]. However, randomized controlled trials (RCTs) are needed to determine the benefit of dexamethasone in neurolisteriosis [66]. An additional issue to analyze is whether adjunctive dexamethasone should be considered in patients with neurolisteriosis on chronic steroid therapy. In this regard, a cohort study assessed 87 cases of C-ABM that occurred in patients using immunosuppressive medication [103]. In this prospective study, *L. monocytogenes* was the second causative organism (40%) after *S. pneumoniae* (41%), immunosuppressive medication consisted of corticosteroids (prednisone) in 82% of cases, and 60% of these C-ABM cases were treated with adjunctive dexamethasone (10 mg QID for 4 days administered before or together with antibiotics) [79, 107]. Mortality tended to be lower in those on adjunctive dexamethasone therapy (19%) as compared to those without adjunctive dexamethasone (34%), and adjunctive dexamethasone did not influence the rates of unfavorable outcomes [107]. These observations may suggest that adjunctive dexamethasone is not associated with harm in patients using immunosuppressive medication who are admitted with bacterial meningitis, including neurolisteriosis [107]. However, not only listerial meningitis patients were involved in this cohort study but also RCT required to validate these findings [107].

### 9.3. Neurosurgery for Listerial Brain Abscess

Neurosurgery may be required for those cases with listerial brain abscess. In a literature review of 73 cases with listerial brain abscess, 52% underwent neurosurgery for drainage or resection, and 45% were treated only with antibiotics [58]. Those cases that underwent neurosurgery showed a lower mortality rate (13%) compared with those that did not undergo surgery (38%). It was also found that a combined approach of neurosurgery and prolonged antimicrobial therapy improved survival. Thus, in those who underwent surgery and were treated with antibiotics for ≤ 6 weeks, the mortality rate was 13%, while in those who underwent surgery and received antibiotics for ≥ 8 weeks, the mortality rate was 0% [58]. Similar data showing higher survival rates in those cases with listerial brain abscess that underwent neurosurgery have been described in other reviews [59, 62, 108]. Although there is no specific guidance for the management of listerial brain abscess, the findings of these literature reviews are consistent with recommendations described in the ESCMID guideline on the treatment of brain abscess [58, 109]. That guideline recommends neurosurgery in all patients with brain abscess whenever feasible, except for cases with toxoplasmosis, and 6–8 weeks of intravenous antimicrobial therapy for aspirated or conservatively treated brain abscesses [109]. Surgical drainage is also suggested for large abscesses (> 2.5 cm) [62]. But, according to a prospective study of 71 cases, many of the listerial brain abscesses observed by neuroimaging can range in size from 7 to 18 mm [70]; therefore, those cases would not require neurosurgery.

## 10. Complications and Outcomes

Neurolisteriosis is a deadly neurological infection associated with poor outcomes and frequent sequelae in surviving patients. Neurolisteriosis mortality ranges from 17% to 35% [5–7, 59, 64, 65, 67, 68, 71, 80, 85]. Likewise, a mortality rate of roughly 35% has been reported for patients with listerial rhombencephalitis [61, 64]. And a higher mortality rate of 25%–44% has been described for listerial CNS abscess [45, 48, 58, 60, 62, 108], which is even higher than that reported for other causes of brain abscess (7%–20%) [45, 60, 62, 109].

The most common causes of death reported for neurolisteriosis include multiorgan failure, cardiac and respiratory failure, and severe brain damage (i.e., seizures and hydrocephalus) [5, 7, 68]. In addition, older age, immunocompromised state (mainly malignancy), focal neurological findings, parenchymal images, inappropriate or delayed antimicrobial therapy, concomitant bacteremia, and previous corticosteroid therapy have been described as factors most commonly associated with neurologic sequelae and death for neurolisteriosis [7, 17, 19, 65, 68–70]. Hence, most neurolisteriosis cases (43%–60%) require management in intensive care units [66, 68]. Between 19% and 33% of patients require mechanical ventilation, and roughly 65% of those patients do not survive [65–68].

Several life-threatening complications have been frequently reported in patients with neurolisteriosis during their hospital stay. Hyponatremia occurs in 41%–80% of cases [7, 8, 64, 65, 67]. Compared to L. *monocytogenes*, this complication has been reported less frequently (26%) for other causes of C-ABM [8]. Seizures have also been observed in 16%–20% of cases during their hospital stay, and it may persist in more than half of cases that presented seizures at admission [65–67, 71]. Furthermore, several studies have found that seizures are an independent risk factor for mortality in neurolisteriosis [65, 73]. Hydrocephalus is a severe complication in roughly 5% of adults with C-ABM [76], whereas 7%–19% of patients with listerial meningitis develop hydrocephalus [5, 7, 65, 66, 70, 71, 77, 78, 82]. Time to presentation (> 4 days) and delaying appropriate antimicrobial therapy may be associated with the presence of hydrocephalus [5, 77]. When initial antimicrobial therapy is inadequately covered for *L. monocytogenes* at admission, hydrocephalus usually occurs several days or weeks after presentation [5, 77, 82]. Once neurolisteriosis cases develop hydrocephalus, they present a greater need for mechanical ventilation (60% vs. 20%) and higher mortality (75% vs. 16%) compared to the absence of hydrocephalus [5].

Without adequate antimicrobial therapy, the neurolisteriosis mortality rate is near 100% within a few days after admission [5, 19, 68, 69]. In a retrospective cohort study including 36 cases with neurolisteriosis, delaying appropriate antibiotic therapy for more than 6 h after admission was associated with a 3-fold increased mortality risk [69]. Of note, the proportion of cases without adequate initial antimicrobial therapy is reported to be high [5, 65–67]. A prospective cohort of 30 adults with neurolisteriosis showed that initial antimicrobial therapy was inadequate in 30% of cases [67]. Another prospective cohort study of 162 adults with community-acquired neurolisteriosis showed that 20% received inadequate initial antibiotic treatment (other than aminopenicillins) [66]. In this cohort study, receiving inadequate antibiotic therapy was due to misdiagnosis with sepsis, pneumonia, or another infection than meningitis in most cases. And outcomes were unfavorable in 80% of the patients initially misdiagnosed and treated with inadequate antibiotics [66]. Likewise, a cohort study of 100 patients with neurolisteriosis showed that empiric treatment was inappropriate in 48% of cases [65]. In this study, the mean delay of proper initial antimicrobial therapy was 7 days, which significantly increased the risk of mortality and development of neurological sequelae [65].

Some radiological signs also suggest poor outcomes in neurolisteriosis. Ventricular involvement (hydrocephalus and/or contrast-enhancing ventricles) and parenchymal imaging (abscess[es], nodule[s], and/or white matter images) have both been associated with five-fold increased mortality for neurolisteriosis [70]. Likewise, a mortality rate of 20% and neurological sequelae of 58% in those who survive have been reported when rhombencephalitis, supratentorial lesions, and brain abscess are the main cranial MRI findings [71].

Long-term neurological sequelae have been observed in 13%–63% of surviving patients with neurolisteriosis, which is higher than that reported for bacterial meningitis (18%) and infectious encephalitis (33%) due to common pathogens [5, 7, 8, 12, 64, 65, 68, 71, 81]. The most common neurological sequelae observed in neurolisteriosis are cerebellar syndrome, limb motor deficiency, cranial nerve palsy, hearing impairment, cognitive impairment, epileptic seizure, tetraplegia, and coma [7, 8, 66–68, 71]. Persistent neurological impairments tend to be higher in those cases with brainstem involvement, in which neurological sequelae have been reported in 45%–63% of surviving patients [5, 64, 65, 68]. The persistence of neurological sequelae in neurolisteriosis may also be related to the existence of neurological impairments upon admission [71]. Fifty to 100% of patients who present with any neurological impairment at admission show persistence of the same impairments during follow-up [71]. Finally, less than half of cases fully recover from the disease. A prospective cohort study including 252 patients with neurolisteriosis reported that almost 40% of them survived and fully recovered without persistent neurological impairments after appropriate treatment [68].

Regarding nosocomial cases, the proportion of those who die among nonpregnant adults with hospital-acquired neurolisteriosis varies from 20% to 100% according to case series and outbreaks reported that include this information [19, 20, 29, 30, 74].

## 11. Conclusion

In high-income countries, *L. monocytogenes* represents the third cause of C-ABM with a low incidence in adults. Neurolisteriosis refers to four clinical syndromes that must be distinguished for a better therapeutic approach. Clinical signs and symptoms of neurolisteriosis can resemble any acute bacterial meningitis due to more common pathogens, but in most cases, it is characterized by clinical meningoencephalitis more than isolated clinical meningitis. Characteristic CSF findings of meningeal involvement tend to be less apparent in neurolisteriosis compared with other causes of bacterial meningitis. Furthermore, atypical CSF findings may be even more frequent when the infection involves focal lesions, as with listerial rhombencephalitis and brain abscess. Likewise, CSF culture yield is adequate to confirm the diagnosis of listerial meningitis and meningoencephalitis; however, it may be deficient in listerial rhombencephalitis and brain abscess, in which case blood culture may improve the diagnostic performance.

Neuroimaging of listerial CNS infection is nonspecific, and different kinds of lesions can coexist in the radiological findings; however, in most cases, neuroimaging can distinguish the main clinical presentations of the disease, which allows for better prognostic accuracy and can also guide the duration of antibiotic therapy. This is more relevant in those cases with listerial rhombencephalitis or brain abscess, which show a higher mortality rate, and more frequent neurological sequelae, and they require prolonged antibiotic therapy until the lesions observed on neuroimaging are resolved. Listerial rhombencephalitis and brain abscess are the least common presentations of neurolisteriosis, but they also pose the most serious problems. The diagnosis of listerial rhombencephalitis can be a challenge, as it has a wide differential diagnosis with other infectious, autoimmune, and paraneoplastic causes of rhombencephalitis; furthermore, unlike most neurolisteriosis cases, listerial rhombencephalitis frequently occurs in young immunocompetent people; its detection by neuroimaging usually requires MRI rather than CT scan, and its microbiological diagnosis has low sensitivity; thereby, these factors could lead to a delayed diagnosis or decrease the level of suspicion. Meanwhile, the main problem in listerial brain abscesses would be the unclear definition of criteria to consider neurosurgery for therapeutic purposes. However, most cases with listerial brain abscess have shown better outcomes with neurosurgery for drainage associated with prolonged antibiotic therapy.

Early administration of antimicrobial therapy with aminopenicillins (or penicillin) is a key to improving outcomes in all suspected or confirmed cases of neurolisteriosis. TMP-SMX is a reasonable alternative for those with aminopenicillin allergy or treatment failure. Other options, such as combination therapies, linezolid, and moxifloxacin, should be considered for those cases where discontinuation of aminopenicillins and TMP-SMX treatment is necessary due to an allergic reaction, toxicity, or therapeutic failure. But experience with these alternative therapies is scarce, and drug safety is of concern.

In addition to early diagnosis and appropriate antibiotic use, the timely use of adjunctive dexamethasone could also improve outcomes. Some cohort studies have already shown favorable outcomes of adjunctive dexamethasone in neurolisteriosis; however, the low incidence of this disease limits the possibility of conducting RCT to confirm these findings.

The definition of hospital-acquired neurolisteriosis requires finding the source of nosocomial infection. Therefore, to confirm its diagnosis, the same genotype and/or serotype of *L. monocytogenes* strains must be isolated from both food served at a hospital and from patients who record consumption of this food and develop meningitis or meningoencephalitis during their hospital stay. The wide range of incubation periods of this disease limits the distinction between community-acquired and hospital-acquired listeriosis. The occurrence of hospital-acquired neurolisteriosis in adults is difficult to state because the available data have been obtained mainly from developed countries, and its confirmation demands highly complex laboratory methods that could be out of reach for many low-income settings. The diagnosis of hospital-acquired neurolisteriosis must be suspected in patients with a history of immunocompromised status or on immunosuppressive therapy and develop acute neurological symptoms during a hospital stay, even if there is no evident source of infection.

Having policies for preparing and providing food to inpatients is crucial, as well as having hospital-based surveillance strategies for preventing further nosocomial cases and outbreaks. Some developed countries apply these elements at local and national levels. In healthcare facilities where these elements are or are not available, healthcare professionals should counsel inpatients at increased risk for listeriosis to avoid foods that may be contaminated by *L. monocytogenes*.

Finally, another concern is the paucity of epidemiological data on neurolisteriosis in developing countries. This neglect also contributes to poorer outcomes in disease prevention in this scenario.

## Figures and Tables

**Table 1:** Summary of virulence determinants involved in *Listeria monocytogenes* pathogenesis.

	Bacterial virulence factor	Target protein	Host-pathogen interaction end result (at intestinal and brain level)
First stage: cell invasion	InlA	E-cadherin receptor	Actin polymerization and cell membrane remodeling of nonphagocytic (epithelial cells) and phagocytic (dendritic cells, macrophages, and monocytes) cells to induce listeria uptake and formation of internalization vacuole
	InlB	Met receptor	

Second stage: Intracellular life cycle	LLO, PC-PLC, and PI-PLC toxins	…	Lysis of listeria vacuolar membrane to release the bacterium into host cell cytoplasm
	ActA	Arp2/3 complex	Polymerization of the actin filament network that creates a comet tail at one pole of the bacterium propelling it toward the host cell surface
	InlC	Adapter protein tuba	Actin recruitment inhibition at host cellular membrane to overcome cortical membrane tension for leaving an infected cell and invading a neighboring cell

*Note:* InlA = internalin A, InlB = internalin B, E-cadherin = epithelial-cadherin, Met = named because gene was discovered after a treatment with N-methyl-N'-nitro-N-nitrosoguanidine, LLO = listeriolysin O, PC-PLC = phosphatidylcholine-specific phospholipase, PI-PLC = phosphatidylinositol-specific phospholipase C, ActA = actin assembly-inducing protein, and InlC = internalin C.

Abbreviation: Arp = actin-related protein.

**Table 2:** Epidemiological features' summary of nosocomial neurolisteriosis cases reported in the last 40 years.

No of cases	Age (years)	Gender	Underlying conditions	Reason for admission	Listerial symptoms onset	Outcome	Outbreak-associated	Possible or proved source of nosocomial infection	Country	Year of infection	Ref.
3	49	Female	Diabetes mellitus	Right foot ulcer and osteomyelitis	Day 7 after admission	Survived	No	NA	Taiwan	2011	[19]
	65	Female	Non-Hodgkin's lymphoma	Cancer management	Day 40 after admission	Dead				2015	
	61	Female	Nephrotic syndrome	Nephrotic syndrome management	Day 22 after admission	Dead				2016	

1	50	Male	Myelofibrosis managed with hydroxyurea	Gastrointestinal bleed (previous hospital stay)	24 h after discharged	Survived	Yes	Ice cream product and milkshake machine	USA	2014	[25, 26]

2	NA						Yes	RTE sausage products and single–food supplier company	Germany	2014–2019	[28]

2	71	Male	Pyoderma gangrenosum, ciclosporin, and steroid therapy	NA	≤ 24 h^∗^	Dead	Yes	Camembert cheese and single dairy	Norway	2007	[29]
	69	Female	Metastatic colon cancer and steroid therapy		4 days^∗^	Dead					

4	Mean age, 67	NA	Cancer, heart failure, renal failure, diabetes, autoimmune disease, or steroid therapy	NA			Yes	RTE scalded sausage and single hospital meet supplier	Germany	2006-2007	[33]

1	51	Female	Crohn's disease, azathioprine, and steroid therapy	Crohn's exacerbation	6 weeks after admission	Survived	Yes	Deli meat products	Canada	2008	[45]

3	24	Female	Acute lymphoblastic leukemia	Chemotherapy	Day 12 after admission	Dead	No	NA	China	1999–2011	[74]
	43	Female	Dermatomyositis, diabetes, and hepatocellular carcinoma	Treatment of underlying conditions	Day 20 after admission	Survived					
	53	Male	Still's disease	Treatment of underlying condition	Day 44 after admission	Survived					

5	46–89	NA	Subarachnoid hemorrhage and steroid therapy	NA	3–140 days after admission	NA	Yes	Raw vegetables (celery, tomatoes, and lettuce), fish, chicken salad, and cheese	USA	1979	[75]
			Aplastic anemia and steroid therapy								
			Biliary obstruction, diabetes, and congestive heart failure								
			Vasculitis and steroid therapy								
			Asthma and steroid therapy								

*Note:* NA = not available (most of the cited references describe consolidate data of different forms of invasive listeriosis, including neurolisteriosis).

^∗^Described as incubation period.

## Data Availability

Data sharing is not applicable to this article as no new data were created or analyzed in this study.
